# The Increased Cardiovascular Risk in Patients Affected by Autoimmune Diseases: Review of the Various Manifestations

**DOI:** 10.14740/jocmr2122w

**Published:** 2015-04-08

**Authors:** Alessandro Durante, Sofia Bronzato

**Affiliations:** a Ospedale Valduce, Como, Italy; b Universita Vita-Salute San Raffaele, Milan, Italy

**Keywords:** Autoimmune diseases, Atherosclerosis, Coronary microvascular dysfunction, CAD

## Abstract

Cardiovascular and autoimmune diseases are among major health concerns in developed countries, and both represent a significant source of morbidity, mortality and economic costs. Despite they are thought to affect subjects at different ages, most of the deaths of patients affected by autoimmune diseases are represented by cardiovascular deaths. Several manifestations of cardiovascular diseases can be observed in patients with autoimmune diseases, such as endothelial dysfunction, accelerated atherosclerosis and an increase in the rate of acute coronary syndromes. Thus, people with autoimmune diseases have an increased cardiovascular risk and a worse outcome in the case of cardiovascular events. In this review, we will describe the correlations between the two spectra of diseases.

## Introduction

Cardiovascular diseases are the main cause of morbidity and mortality and autoimmune diseases are among the leading causes of death among young and middle-aged women in developed countries [1]. Despite these two spectra of disease apparently affect people at different ages, autoimmune diseases can cause endothelial dysfunction, accelerated atherosclerosis and an increase in the rate of acute coronary syndromes due to inflammation, endothelial dysfunction and also secondary to the drugs administered to control the immune process. We will review the relationships between autoimmune diseases and various manifestations of cardiovascular disease.

## Accelerated Atherosclerosis

Inflammatory and autoimmune diseases, such as rheumatoid arthritis (RA) and systemic lupus erythematous (SLE), suffer from increased cardiovascular morbidity and mortality owing to accelerated atherosclerosis and premature coronary artery disease [2, 3], and this is especially evident in young women.

Systemic sclerosis (SSc), an autoimmune disease with a less prominent inflammatory component but significant vascular abnormalities [4], has also been proposed to cause premature atherosclerosis as well [5-7]. Two meta-analyses of studies assessing subclinical atherosclerosis showed that SSc patients had significantly increased carotid intima-media thickness (IMT), indicating an increased atherosclerotic burden, as compared with controls [8, 9]. In general, the prevalence of traditional cardiovascular risk factors does not seem to be increased in SSc [10]. Blood pressure has been shown to be similar between SSc and controls [11]. The frequencies of obesity, hyperlipidemia, hypertension, and diabetes were not increased in SSc [6, 10, 12]. Still, other disease-specific factors such as medication use and lack of exercise may be contributing factors to atherosclerotic disease.

Many observational studies in cohorts of RA patients with different characteristics reported a wide range of incidence rates for cardiovascular events and mortality. Some studies show a more than doubled risk compared to general population, which is comparable to the cardiovascular risk in diabetes mellitus [13, 14], while other studies show a more modest increased risk [15]. However, a pooled analysis showed that the risk of cardiovascular events and mortality is approximately 50% higher in RA patients compared with the general population [16]. The traditional risk factors, which can cause and predict cardiovascular morbidity and mortality in the general population but also in people with diabetes, seem to be equally or only slightly more prevalent in the RA population. Some risk factors, such as smoking, even play an important role in the onset of RA. However, traditional risk factors alone do not explain the higher cardiovascular risk in RA [17]. At present, RA and atherosclerosis are both regarded as inflammatory-linked diseases. Many epidemiological, clinical, and laboratory investigations have suggested that the chronic inflammation and immune dysregulation in RA have a key role in accelerating atherosclerosis [18-20].

Another factor that may link atherosclerosis to RA is a common genetic background. An increasing number of studies report gene polymorphisms that are associated with a higher risk of cardiovascular diseases in RA [21]. Human leucocyte antigen shared epitope (HLA-DRB1) is a typical example of a gene that seems associated with cardiovascular risk in RA [22].

However cardiovascular risk is increased before the clinical onset of RA. In fact several studies showed that the first signs of atherosclerosis are already present in patients with recent-onset RA [23], with significantly higher carotid IMT. Moreover, these early signs of atherosclerosis have been found to be correlated with systemic inflammatory markers and disease severity markers [24].

Over the past few decades, the survival rate for patients with lupus has improved from 50% to between 80% and 90% at 10 years. However, the mortality rate for patients with SLE is three times that in the general population [25]. Most of this mortality is attributed to premature coronary atherosclerosis. Autopsies have shown that coronary atherosclerosis is present in up to 40% of patients with SLE, as compared to 2% of matched controls [26].

A review by Karrar et al found that the prevalence of coronary artery disease among women aged 35 - 44 years with SLE is 50 times higher than among age- and sex-matched controls [27].

Multiple mechanisms account for the increased atherosclerotic burden in this spectrum of diseases. In previous studies, an abnormal lipid profile was reported in 46-56% of SLE patients [28]. Renal involvement with nephrotic syndrome is frequent in autoimmune diseases and can lead to hyperlipidemia.

Cardiovascular diseases are chronic diseases associated with an inflammatory component and inflammation is considered a novel risk factor for coronary heart disease with growing importance. Despite still debated, this inflammatory component is currently seen as not directly induced by an autoimmune process. A non-autoimmune primary pathological process such as apolipoprotein B-containing lipoproteins deposition may result in a vicious cycle of inflammation promotion and perpetuation. Subendothelial retention of apolipoprotein B-containing lipoproteins triggers a maladaptive, non-resolving inflammatory response that drives atherogenesis [29]. A systemic inflammatory disorder may increase and accelerate the response to this injury, thus leading to accelerated atherosclerosis. A pathological study showed that coronary vasculitis and immunologic injury secondary to immune complexes deposition may be responsible for the development of coronary disease in patients with SLE [30]. In fact previous immunopathologic studies demonstrated immune reactants in the walls of inflamed and non-inflamed arterial segments in a pattern consistent with immune complex aggregates.

In addition, chronic steroid therapy (a mainstay of treatment for SLE) worsens hyperglycemia and hyperlipidemia, contributing to atherosclerosis. The role of corticosteroids in the atherosclerosis of SLE has not been fully defined. It has been argued that steroids may be a double-edged sword, such that at low dosages, they may exert beneficial anti-inflammatory effects, while at high dosages, steroids may cause harm by exacerbating metabolic cardiovascular risk factors [28, 31].

Two previous studies of subclinical atherosclerosis in SLE showed that long-term treatment with corticosteroids was not associated with a significantly increased risk [32, 33]. Indeed, Roman et al [33] found that patients without carotid plaque disease received a significantly higher mean daily dose of prednisolone and more of them had taken cyclophosphamide as compared with patients with carotid plaque disease, suggesting that more aggressive control of disease activity might prevent atherosclerosis.

Besides glucocorticoids, other immunosuppressive agents have been implicated in the pathophysiology of atherosclerosis [34]. Azathioprine can be hepatotoxic and may induce fatty liver, with overflow secretion of very low-density lipoprotein cholesterol, and can induce a secondary mixed hyperlipidemia in susceptible patients.

Cyclosporin A can increase low-density lipoprotein (LDL) cholesterol levels and induce glucose intolerance, both of which can cause atherosclerosis. In addition, cyclosporin A has been shown to be associated with endotheliitis [35], increased circulating levels of endothelin 1, and coronary microvascular dysfunction (CMD) [36].

It is worth noting the possible positive and adverse effects of anti-inflammatory drugs in atherosclerosis. In the case of acute myocardial infarction (MI), the rate of recurrence is higher in the first few months. This heightened risk is at least in part driven by a post-MI monocytosis. The inhibition of this monocytosis might reduce the risk of recurrent events [37] but it also may delay tissue repair of the infarcted myocardium itself [38], thus leading to possible mechanical complications such as heart rupture.

In addition, patients with autoimmune diseases often have antiphospholipid syndrome, predisposing them to thrombosis and MIs. The literature also reports patients treated for spontaneous coronary artery dissections secondary to SLE [39]. Other potential mechanisms involved in the pathophysiology of coronary artery disease in lupus include microvascular disease and coronary aneurysms. Active lupus can cause coronary vasculitis and rarely may lead to MI [7]. ROS oxidizes LDL, and the subsequent consumption of oxidized LDL (ox-LDL) by monocytes is what leads to the formation of atherogenic foam cells [40]. Although foam cells have not been studied in SSc, circulating ox-LDL/β2-glycoprotein 1 complexes [41] and anti-ox-LDL antibodies may be elevated in SSc patients [42]. SSc patients may also have higher levels of atherogenic proinflammatory high-density lipoprotein and lipoprotein (a) [43].

## CMD

Recent studies demonstrate that, in addiction to epicardial vessel plaques, also CMD can be detected in patients affected by chronic inflammation disorders [25]. The clinical significance of microvascular dysfunction lies in its association with worse cardiovascular outcomes [44].

Previous studies demonstrated diffuse microvascular dysfunction in SLE. This is caused by increased sympathetic outflow [45] and increased endothelin-1 [46] in SLE, both of which are constricting factors and could influence flow mediated vasodilation (FMD). Another contributor to the difference in FMD may be a reduced bioavailability of NO in response to shear stress. This may be as a consequence of reduced production of NO or alternatively increased destruction. Also increased oxidative stress may account for the endothelial dysfunction in SLE. Oxidative stress produced by the interaction between superoxide and NO may be of particular importance in relation to vascular functions of NO [47]. Superoxide (O_2_-) combines almost instantaneously with NO to form peroxynitrite that has a dual effect of decreasing NO bioactivity while promoting protein and lipid oxidation [48] and both O_2_- and peroxynitrite have been shown to be increased in SLE [49].

Cardiac disease is a common finding also in SSc even if it is often clinically occult. In fact, myocardial disease is evident in 20-25% of patients with SSc, while at post mortem pathologic studies report a myocardial involvement in up to 80% of patients [50, 51]. The primary cardiovascular disorder in SSc is CMD with diffuse arteriolar and capillary lesions that precede myocardial fibrosis. The mechanisms of endothelial and microvascular dysfunction in SSc have been explored in the previous paragraph.

Coronary flow reserve (CFR) has been considered a useful diagnostic index for the functional and physiologic assessment of coronary circulation [52]. The CFR represents the ability of coronary blood flow to increase above its basal level when the coronary vascular bed is maximally dilated. It is a global parameter of coronary blood flow, which is altered in the presence of epicardial coronary artery stenosis, and, in the absence of epicardial stenosis, it will reflect abnormalities of the coronary microcirculation [53].

CFR has been demonstrated to be impaired in most patients with SSc, even in the absence of clinical signs of cardiac disease [54]. CFR impairment in patients with SSc is likely to indicate an anatomical and/or functional impairment of coronary microvasculature because the presence of structural impairment of small coronary arteries has been pathologically demonstrated in these patients in the absence of abnormalities of epicardial vessels [55].

CMD can be also detected in RA. There is accumulating evidence that cardiovascular risk is increased before the clinical onset of RA. Several studies showed that endothelial dysfunction can be detected in patients with recent-onset RA [23]. Gonzales-Gay et al [21] reported endothelial dysfunction in 10 newly treated patients with RA that received intrabrachial artery infusions of acetylcholine. They also reported the presence of endothelial dysfunction in long-standing RA patients receiving standard methotrexate therapy. Similar findings were observed in recent studies using FMD technique to evaluate endothelial function. Furthermore, endothelial dysfunction was also found in a series of 32 young to middle-aged RA patients without CV risk factors and low disease activity [56]. A study by Ciftci et al showed an impaired CFR in 30 RA patients compared to healthy controls [57].

There is an additional concern related to CMD. The most impressive expression on CMD is the occurrence of no reflow (NR). NR is responsible for the inability of a previously ischemic region in the territory of a recanalized infarct-related artery to be reperfused. NR is caused by functional and structural damage [58] of coronary microvasculature during ischemia [59]. This damage is aggravated when IRA is reperfused [60].

Different pathogenetic mechanism predispose to NR. First there could be an irreversible damage of cellular components of microvascular circulation in the ischemic area when flow is restored. In fact prior to cell death there is a period during which the ischemic myocyte is viable, but vulnerable to further injury if blood flow is restored. Reperfusion causes the reintroduction of oxygen and energy into an abnormal cellular environment and triggers additional events that produce further myocyte damage. Either there could be a functional impairment of the microvascular coronary circulation with myocyte hypercontracture [61], platelet activation [62], loss of endothelium mediated vasomotion, alteration of sympathetic innervation, and external compression owing to interstitial edema.

The presence of a pre-existing CMD poses patients affected by autoimmune diseases to an increased risk of NR occurrence. It is worth noting that NR occurrence is strongly related to a worse prognosis [63].

Despite the association between autoimmune disease and NR has not been addressed in the literature, in our experience these patients often show NR after both primary and non-primary percutaneous coronary revascularization, with even increased rate after stent deployment.

## Myocardial Infarction and Coronary Revascularization

Studies from the general population demonstrate association between markers of inflammation and increased levels of matrix metalloproteinases, markers of oxidant stress, and tissue factor, all of which are purported to have a role in plaque destabilization and thrombosis [64-66]. Patients with autoimmune diseases have an increased risk of cardiovascular events and adverse cardiovascular outcomes. Also the outcome of patients affected by autoimmune diseases after coronary revascularization can be expected to be worse compared to the general population, and similar to other high risk patients such as diabetics.

A previous study showed a 50 times greater of MI in women affected by SLE aged 35 - 44 compared to women of similar age from a population-based sample [3]. Although coronary disease is the leading cause of death among women, coronary events occur rarely in women under the age of 55 years [67]. In contrast to the general female population, in this study 67% of women under the age of 55 had their first cardiovascular event.

Urowitz et al suggested for the first time that SLE mortality followed a bimodal pattern. In fact, whereas deaths early in the course of the disease are most often due to active lupus, later deaths are frequently secondary to atherosclerotic coronary heart disease and acute MI [68].

Although Ward et al found similar in-hospital outcomes for MI between SLE and non-SLE patients, the only systematic study examining coronary artery bypass grafting (CABG) in patients with connective tissue diseases found lower survival and a higher frequency of reintervention (mean follow-up: 35 months) when compared to the general population [69, 70].

A recent study on SSc patients showed that they had a significantly increased risk of MI and stroke after a mean follow-up of 5.5 years, with a hazard ratio of 1.97 for MI and 2.56 for stroke [71]. In this study patients had lower frequencies of overweight and obese status, more frequent past smoking status, and more frequent use of aspirin, non-steroideal anti-inflammatory drugs and oral glucocorticoids. However, as previously discussed, patients with SSc do not have higher frequencies of traditional cardiovascular risk factors but therapy may be implicated in the increased risk. The increased risk of cardiovascular events in SSc may be due to atherosclerosis. Alternatively, these events may represent the effects of vasospasm, SSc specific vasculopathy, vasculitis, thrombosis or a combination of atherosclerotic and non-atherosclerotic factors.

A population-based cohort study of RA patients reported an increased risk of coronary heart disease and MI 2 years prior to RA diagnosis using the American College of Rheumathology 1987 criteria [72]. Almost half of all deaths in RA result from cardiovascular causes, which is predominantly due to MI or congestive heart failure [73].

In addition antithrombotic therapies are less likely to be prescribed at discharge in patients with autoimmune diseases as reported by Maksimowicz-McKinnon et al [74]. Lower adherence to statin prescription for secondary prevention in SLE patients was evident in the same study.

## Conclusion

Autoimmune diseases predispose to accelerated atherosclerosis and various manifestations of cardiovascular diseases. It is therefore of main importance in these patients to have cardiovascular risk factor assessment and to manage optimally known risk factors. Moreover screening for cardiovascular diseases should be performed in advance in patients affected from autoimmune diseases, since cardiovascular deaths are among the main causes of mortality in these patients.
